# How to manage missing covariates in randomized controlled trials: a comparison of strategies

**DOI:** 10.1186/s12874-025-02708-w

**Published:** 2025-11-25

**Authors:** Shiyu Zhang, Yajuan Si, John J. Dziak

**Affiliations:** Institute for Social Research, University of Michigan, 426 Thompson St, 48104 Ann Arbor, MI US

**Keywords:** Randomized controlled trials, Missing covariates, Multiple imputation, Grand mean imputation, Missing indicator method

## Abstract

**Background:**

When analyzing randomized controlled trials (RCTs) data, covariate adjustment is often employed to increase the precision of estimated treatment effects. Missing data in covariates, if not handled properly, can result in biased and inefficient estimates. However, the existing literature on handling missing covariate data is limited, and recommendations vary regarding a valid and efficient approach.

**Methods:**

To help reconcile the seemingly inconsistent recommendations, we address two questions through methodological descriptions and simulated demonstrations. First, how should a multiple imputation (MI) model be specified for RCTs to best preserve the benefit of the randomization design? We consider three different approaches: MI with only baseline variables, “MI overall”, and “MI by arm”. Second, when and why will simple general strategies, such as grand mean imputation and the missing indicator method, perform as well as or better than MI in estimating treatment effects, and when and why do they fail?

**Results:**

“MI by arm” has the potential to produce unbiased estimates for both the average and subgroup treatment effect (primary and secondary analyses) under the missing at random assumption. Strategies that capitalize on the randomization design, including MI with baseline variables, grand mean imputation, and the missing indicator method, may generate unbiased estimates for the average treatment effect (primary analysis) regardless of the missing data mechanism.

**Conclusion:**

This article clarifies the assumptions and mechanisms by which different missing data strategies accommodate missingness in covariates and reconcile recommendations that sometimes appear contradictory in the literature. Under MAR, “MI by arm” produces unbiased estimates for both the average treatment effect and subgroup treatment effects. Leveraging the randomization design, “baseline-only MI”, grand mean imputation, and the missing indicator method produce unbiased estimates for the average treatment effect, but biased subgroup treatment effects, regardless of the missing data mechanism.

## Background

Covariate adjustment is common in the analysis of randomized controlled trial (RCT) data. For example, our systematic search on PubMed in 2025 May identified 77 RCTs published between 2024 and 2025 in the Journal of the American Medical Association, 45 of which reported treatment effects adjusted for covariates. When covariates are prognostic of the experimental outcome, adjusting for covariates improves the precision (i.e., reduces the variance) of the estimated treatment effect (see1for details, 2–5). However, missingness in covariates in RCTs remains an issue that is not adequately addressed. Most methodological guidelines about missing data in RCTs focus on missingness in outcomes (e.g., 6, 7, 8), and they might not apply to missing covariates. Among the 45 RCTs that reported adjusted effects, only 10 explained how they anticipated and managed missing data in covariates, and there was no consensus on the recommended strategies.

Missingness in covariates should not be neglected. If the list of covariates is long, even a sparse missingness pattern can result in a noticeable portion of incomplete cases [1]. If one were to remove subjects with missing covariate values from the analysis, reduction in sample sizes would diminish the precision of treatment effect estimates. Additionally, unless missingness is missing completely at random (MCAR), removing subjects may also result in biased treatment effect estimates; this is particularly concerning when the treatment effect varies across subgroups and the subgroup composition in complete cases does not accurately reflect the intended experimental sample.

In practice, missing data often occur in both the outcome and covariates; however, compared to outcomes (e.g., 6, 7, 8), much less attention has been given to clarify the assumptions and mechanisms by which missingness in covariates can be addressed, particularly in the context of RCTs. Notably, missingness in covariates raises separate issues compared to missingness in the outcome and requires different guidelines from those managing missing outcomes. To illustrate, if missing data occur in the outcome only, even a complete-case analysis, as the default option in most statistical software, could be sufficient for obtaining unbiased estimates, provided that the outcome is *missing at random* (MAR) given the covariates (i.e., the included covariates can account for the correlation between the missingness and the outcome) [2–5]. However, if missing data also occur in covariates, complete-case analysis only yields unbiased estimates in general if covariates are *MCAR* (i.e., the probability of missingness in covariates is the same for all cases) (see 9, 13 for details). If investigators were to make a less restrictive and more realistic MAR assumption for covariates, then other strategies need to be applied to manage missingness in covariates to prevent biased inferences and loss of statistical power. This is to illustrate the fact that missingness in covariates presents unique analytic challenges in statistical modeling that differ from and extend beyond those caused by missingness in the outcome [1, 5, 6].

In the literature, strategies for handling incomplete covariates were originally developed based on observational studies [1, 7, 8], but RCTs represent a special case where strategies and assumptions from observational study data are not always directly applicable in the same way. Considerations for multiple imputation (MI) differ between observational studies and RCTs, especially with respect to missingness in covariates; simply “applying MI” is not a panacea. This is the case for at least three reasons.

First, RCTs are typically designed to answer questions about treatments [9, 10]; hence, the missing data strategy should prioritize the estimation of treatment effects, rather than treating all coefficients as being of substantive interest as is sometimes the case in observational studies. We focus on two common estimands in RCTs [9]; an estimand refers to the target quantity that a study aspires to measure [11]. (1) The *average treatment effect (ATE)* quantifies the overall difference between experimental conditions on a prespecified primary outcome and is typically the primary aim of an RCT. (2) *Subgroup treatment effects* capture the heterogeneity of the treatment effect, support the development of individualized treatment regimes, and are often specified as secondary aims.

Second, the randomization design establishes pivotal assumptions for data analysis. Capitalizing on the randomization—i.e., using the reasonable assumption that confounding factors are balanced across arms (in expectation)—the ATE in an RCT could be estimated without bias using more than one possible model. In the simplest form, the ATE can be estimated as the mean difference between experimental arms in the outcome. Covariate adjustment is not strictly necessary, because bias is removed (at least in a large-sample sense) by the randomized design, and covariates are included mainly to improve the precision of the ATE estimate.

Third, since the benefits of RCTs are based on the fundamental assumption that randomization balances confounders across experimental arms, the missing data strategy for incomplete covariates in RCTs should strive to maintain the independence between the treatment assignment and covariates. This is in contrast with observational studies, in which covariates are often potential confounders that are correlated with the treatment or exposure. Given these considerations, recommendations derived from observational studies are not always applicable to RCT data. It is crucial to emphasize the differences in RCTs from observational studies and clarify potential confusions.

### Our contributions

This paper discusses the advantages and disadvantages of different strategies handling missing covariates by outlining the assumptions they rely on, the consequences when their assumptions are violated, and their feasibility of implementation, all related to the specific estimand of interest. Specifically, we consider three MI approaches [12–14] and two simple general strategies, grand mean imputation (GMI) and the missing indicator method (MIM) [15–18]. Because the appropriate assumptions and research questions vary from study to study, we do not assert one strategy as superior to the others. Instead, we clarify the mechanisms under which different missing data strategies can lead to unbiased estimates, focusing on how to benefit the most from the randomized study design. The goal is to support researchers in rigorously evaluating missing data strategies for covariates and help reconcile recommendations that sometimes appear contradictory in the literature. We conduct simulation studies to illustrate the conceptual discussions.

Although all considered strategies have been investigated in both observational and experimental studies, recommendations diverge regarding their validity and efficiency. MI is generally regarded as the state-of-the-art in observational studies [19–22], but there is debate on how best to specify plausible imputation models under various missing data mechanisms [23–29]. In contrast, when estimating ATEs in RCT settings, some simple general strategies that are considered inappropriate in observational studies [8, 19–21, 30] may be practical and efficient for handling incomplete covariates [4, 15–18], as they leverage the ability of randomization to balance covariates.

We address two issues. The first is how to specify MI models for covariates while taking into account the randomization design. We consider three approaches to imputing missingness in covariates: MI with only baseline variables, MI including the outcome in the imputation model fitted with the overall sample (MI overall), and MI including the outcome by randomization arm (MI by arm) [4, 17, 31]. Previous studies have compared MI overall and MI by arm, but their findings and suggestions are inconsistent [4, 17, 31]. In this work, we clarify the underlying assumptions of these perspectives regarding the randomization design, data-generating mechanism, and missing data mechanism and specify the conditions under which they are appropriate.

The second is how to choose missing data strategies for different estimands, specifically average versus subgroup treatment effects. Simple general strategies such as GMI and MIM can produce an unbiased and precise ATE estimate, sometimes even as a preferred alternative to MI [4, 15, 17, 18], but should not be used to estimate subgroup treatment effects. The applicability of these strategies to RCTs may be unexpected for readers familiar with their use primarily in the observational studies, where they are generally regarded as inferior to MI in nearly all scenarios. We will elaborate on the reasons for this perception in the following sections.

Section 2 introduces the setup and notation. Section 3 describes MI and three imputation models for covariates. Section 4 discusses GMI and the MIM. Section 5 presents a simulation study evaluating statistical properties of these missing data strategies. Section 6 concludes with a summary and discussion of the advantages and disadvantages of the different strategies for handling incomplete covariates in RCTs.

## Methods

### Setup

Consider a hypothetical experiment that randomly assigns the treatment to participants, where $\:T=1$ indicates the active treatment arm and $\:T=0\:$indicates the control arm. There are two covariates $\:X=({X}_{1},{X}_{2})$, where $\:{X}_{1}$ is fully observed but$\:\:{X}_{2}$ contains missing values, indicated by a missingness indicator $\:{M}_{2}$. We assume that covariates are measured at or before the time of treatment. Let $\:Y$ denote the continuous experimental outcome and$\:\:Y\left(1\right)$ and $\:Y\left(0\right)\:$denote the potential outcomes for a participant under the treatment and control, respectively.

Using the potential outcome notation, the ATE is defined as: $\:E\left[Y\left(1\right)-Y\left(0\right)\right].$ A randomization design typically meets assumptions including the Stable Unit Treatment Value Assumption (SUTVA; each participant’s outcome depends only on their own treatment assignment, with no interference or hidden variations of treatment), exchangeability (treatment and control arms are comparable), and positivity (each participant has a non-zero probability of being assigned to any arm) [32]. In a randomized study, the ATE can be estimated unbiasedly simply as the mean difference between arms: $\:E\left[Y|T=1\right]-E\left[Y|T=0\right],$ which can be alternatively estimated using a regression model: $\:E\left(Y\right)={\:\beta\:}_{0}+{\:\beta\:}_{1}T$, where $\:{\:\beta\:}_{1}$ is an estimator of the ATE.

Such unadjusted estimation approaches, while unbiased, may not be efficient. Covariate adjustment may improve efficiency by explaining the outcome variability. With covariate adjustment, the *primary analysis model* for the ATE can be written as:

$$\:E\left(Y\right)={\:\beta\:}_{0}+{\:\beta\:}_{1}\:T+{{\:\beta\:}_{2}X}_{1}+{{\:\beta\:}_{3}X}_{2}$$.

Again, the coefficient $\:{\:\beta\:}_{1}$is an estimator of the ATE.

When investigators are interested in examining treatment effect heterogeneity, they focus on subgroup treatment effects, defined as a conditional average treatment effect: $\:E\left[Y\left(1\right)-Y\left(0\right)|X=x\right].$ As above, using the same key assumptions (SUTVA, exchangeability, and positivity), this effect can be estimated as the mean difference between arms conditional on covariates: $\:E\left[Y\left(1\right)|T=1,X=x\right]-E\left[Y\left(0\right)|T=0,X=x\right]$. The estimation can also be performed using a regression model. In our setup, the *secondary analysis model* is specified as

$$\begin{aligned}\:E\left(Y\right)&={\:\beta\:}_{0}+{\:\beta\:}_{1}T\\&+{{\:\beta\:}_{2}X}_{1}+{{\:\beta\:}_{3}X}_{2}\\&+{{\:\beta\:}_{4}X}_{1}T+{{\:\beta\:}_{5}X}_{2}T\end{aligned}$$.

Coefficients$\:{\:\beta\:}_{1}$, $\:{\:\beta\:}_{4}$, and $\:{\:\beta\:}_{5}$ parameterize the subgroup treatment effects; specifically, for given covariate values of $\:{X}_{1}$ and $\:{X}_{2}$,$\:{\:\beta\:}_{1}+{{\:\beta\:}_{4}X}_{1}+{{\:\beta\:}_{5}X}_{2}$ estimates the subgroup treatment effect.

It is crucial to distinguish the substantive analysis model (used to analyze the data and answer research questions), the imputation model (used to impute missing data, if any), and the true underlying model (i.e., the unobserved population or data-generating mechanism). Here we assume that the data-generating mechanism involves a heterogeneous treatment effect in the target population; i.e., the true treatment effect varies across levels of $\:{X}_{1}$ and $\:{X}_{2}$:

$$\begin{aligned}\:Y&={\:\beta\:}_{0}+{\:\beta\:}_{1}T+{{\:\beta\:}_{2}X}_{1}\\&+{{\:\beta\:}_{3}X}_{2}+{{\:\beta\:}_{4}X}_{1}T\\&+{{\:\beta\:}_{5}X}_{2}T+\epsilon\:\end{aligned}$$.

We make two remarks about our specifications of true data-generating model and the analysis models. First, recognizing treatment effect heterogeneity differentiates this work from some other studies that assumed a homogeneous true treatment effect (e.g., 12, 23, 24), which we consider to be limited in real-world complexities [33, 34]. The fact that the true treatment effect might be heterogenous means that simply excluding cases with missing covariates will result in biased ATE estimate. This is because the exclusion could change the subgroup composition in the analytic sample, so the estimated treatment effect, which is essentially a (weighted) average of subgroup treatment effects, no longer reflects the effect in the original randomized sample. Missingness in covariates must be properly dealt with to avoid biased inferences [18]. However, if we had assumed that that the true RCT treatment effect is homogenous (i.e., coefficient of $\:T$ is a constant), the treatment effect can be unbiasedly estimated from any random subset of the sample, so that the choice of a missing data strategy would affect only the estimate variance and not the bias. Second, acknowledging the heterogeneity of the true treatment effect is not in conflict with estimating an ATE. In our setup, secondary analysis model matches the data-generating mechanism by including all relevant interactions, but the primary analysis model is intentionally set as simpler and omits interaction terms. This is common in practice. When estimating the ATE, researchers may prefer parsimonious models without higher-order interactions for better interpretability and stability. Additionally, there simply might be too many covariates for all interactions to be included in the model, whereas the investigator should still acknowledge that the interactions may exist and assess model fit.

### Multiple imputation 

The MI procedure can be divided into three steps [12, 13]. The first step is “imputation”, where MI fits models to the observed data, draws model parameters from their posterior distributions and then imputes draws from a posterior predictive distribution or using predictive mean matching. The result is *M* completed datasets, each with different draws or imputations of the missing values. The standard application of MI works under the MAR assumption, which allows the imputation model estimated on the observed data to approximate the true relationships among variables as if data were complete [6].

The second step is “analysis”, where the substantive model of interest is fitted to each “completed” dataset. In our illustrative example, the following two substantive analysis models are fitted to address the primary aim and the secondary aim of the RCT, respectively. Let $\:{{X}_{2}}^{MI}$ indicate the imputed $\:{X}_{2}$ within each of the imputed datasets.

Primary Model: $$\:Y={\:\beta\:}_{0}+{\:\beta\:}_{1}\:T+{{\:\beta\:}_{2}X}_{1}+{{\:\beta\:}_{3}{X}_{2}}^{MI}+\epsilon\:$$

Secondary Model : $$\begin{aligned}\:Y&={\:\beta\:}_{0}+{\:\beta\:}_{1}T+{{\:\beta\:}_{2}X}_{1}\\&+{{\:\beta\:}_{3}{X}_{2}}^{MI}+{{\:\beta\:}_{4}X}_{1}T\\&+{{\:\beta\:}_{5}{X}_{2}}^{MI}T+\epsilon\:\end{aligned}$$.

(Note that in this simple illustrative example, it would have been feasible to fit only the more complicated model and use it to answer both the primary research question about average effects and the secondary question about interactions. However, recall that in more complicated real-world scenarios, it may not be feasible to include all possible interactions.)

The third step is “pooling”, where the multiple sets of results are combined using Rubin’s combining rules [13] into a single point estimate and variance that account for imputation uncertainty.

Step 2 is guided by investigators’ substantive research questions, and step 3 is a direct application of formulas, making both relatively straightforward. The main challenge is step 1, the appropriate modeling of relationships between observed variables [14]. Generally, imputation models should preserve relationships among variables that will later be used in substantive analyses, a principle known as congeniality [35]; but in practice, this can be complicated to fulfill and may sometimes seem to be in conflict with other modeling goals.

For RCT, it is important to impute covariates in a way that reflects the randomization design, but there is a lack of consensus over how to specify the imputation model for this. The literature presents three broad approaches [4, 17, 31, 36], which we discuss in the next three subsections, respectively.

#### Multiple imputation with only baseline variables

RCTs can be viewed as containing two separate stages: the design stage, when the trial is conceptualized and covariates are measured, and the analysis stage, when the experimental outcome is observed and analyzed [31, 37]. The design stage occurs before the randomized treatment and the analysis stage occurs afterward. It has been argued that the imputation procedure should maintain this temporal separation in order to preserve the direction of the causal pathway. That is, because the outcome was not yet collected at the time when the covariates were observed, and outcome consequently cannot affect the covariates’ values, the imputation model of covariates should use only baseline variables and not involve the experimental outcome. In our illustrative example, this perspective suggests specifying the imputation model of $\:{X}_{2}$ as simply $\:{X}_{2}={{\gamma\:}_{0}+{\gamma\:}_{1}X}_{1}\:+{\epsilon\:}_{2}$, and not using *Y* in the imputation model; we refer to this approach as “baseline-only MI”.

However, it is important to note that while baseline covariates logically cannot be causally influenced by the end-of-study outcome, this does not preclude them from being *correlated* with the outcome. The imputation model aims to capture dependency structure based on all observed information, which may not necessarily follow causal pathways. A problem with baseline-only MI is that associations between the incomplete covariates and the outcome are not explicitly accounted for [31]. This approach contradicts the rationale for covariate adjustment: covariates improve estimation efficiency exactly because they are related to the outcome. In our example, the $\:{X}_{2}$ values generated by baseline-only MI are spuriously forced to be uncorrelated with $\:Y$ conditional on $\:{X}_{1}(\text{i}.\text{e}.,\:Y\perp\:{{X}_{2}}^{imputed}|{X}_{1})$; using them in subsequent analyses will result in biases in any effects related to covariates (e.g., coefficients of interactions between covariates and treatment). Hence, if one wants to correctly model the distributions of covariates and support inferences about effects related to covariates, the outcome should not be omitted even when imputing baseline covariates.

Although baseline-only MI mis-specifies the model for covariates, it offers certain advantages. In particular, the estimated ATE (i.e., coefficient of $\:T$) is unbiased. This is because by using only baseline variables in the imputation model, the imputed values of covariates are ensured to remain independent of the randomized assignment (i.e., $\:\left({X}_{1}{,X}_{2}\right)\perp\:T$) and hence, do not bias the ATE estimation.

#### Multiple imputation overall

Given the limitations of using only baseline variables, an alternative is to include the experimental outcome and the treatment indicator for imputing covariates: $\:{X}_{2}={\gamma\:}_{0}+{\gamma\:}_{1}{X}_{1}+{\gamma\:}_{2}T+{\gamma\:}_{3}Y\:+{\epsilon\:}_{2}$. We refer to this model as “MI overall” following previous studies, because a single model is applied to the entire sample; this is contrast with “MI by arm”, discussed later, where separate imputation models are specified for each experimental arm [4, 17, 31, 36]. With some extra specification, the overall model could include interaction among $\:{X}_{1}$, $\:\:T,$ and $\:Y$. However, we focus on the model specification with only main effect terms; in practice, when an RCT dataset is passed to the widely used R package *mice* without modification, the default model specifies only main effects. This is the result of *mice* implementing chained equations to impute each variable conditional on all other analysis variables [38] although not necessarily on their products. In a richer example with many covariates, there may be many potential interactions, and it might not be convenient or feasible to include them all.

The use of $\:Y$ (a post-randomization outcome) to predict $\:{X}_{2}$ (a baseline covariate) may seem counterintuitive, especially given the general advice not to condition on post-randomization variables when estimating a causal effect of treatment. However, we are considering the imputation model here (i.e., step 1 of MI), not the substantive analysis model (i.e., step 2 of MI); the imputation model is not used to generate causal interpretations.

It has been argued that “MI overall”, compared to “MI by arm”, better respects the randomization design, because MI overall recognizes that covariate distribution should be comparable in the two arms, and hence, a single, pooled model is needed to impute covariates [17]. However, a major limitation of MI overall has not been adequately addressed in prior research: the imputation model of covariates can be *incompatible* with the data-generating model of the outcome, except under a special condition where the treatment effect is truly homogeneous across subgroups. Formally, an imputation model of covariates (a conditional model of $\:X$) and an outcome model (a conditional model of $\:Y$) are said to be incompatible if no joint model exists whose conditionals match these two conditional models (“compatibility” is related to, but not the same as “congeniality;” see [2, 25] for details). Incompatibility can arise when the outcome model is non-linear (e.g., a logit link function) or contains non-linear (e.g., quadratic) or interaction terms [2, 25]. Applying this concept to “MI overall” in our example, the problem is that the implied conditional model for a covariate ($\:{X}_{2}={\:\gamma\:}_{0}+{\gamma\:}_{1}{X}_{1}+{\gamma\:}_{2}T+{\gamma\:}_{3}Y+{\epsilon\:}_{2}$) cannot generate imputed values (of $\:{X}_{2}$) that correctly account for the underlying, different relationships between the covariate and the outcome ($$\begin{aligned}\:Y&={\:\beta\:}_{0}+{\:\beta\:}_{1}T+{{\:\beta\:}_{2}X}_{1}\\&+{{\:\beta\:}_{3}X}_{2}+{\:\beta\:}_{4}{X}_{1}T\\&+{{\:\beta\:}_{5}X}_{2}T+\epsilon\:\end{aligned}$$).

When the imputation model of a covariate is incompatible with the outcome model (i.e., not capturing the treatment effect heterogeneity), both *the ATE* and subgroup treatment effect estimates can be biased. The biases arise because this mis-specified imputation model can induce spurious dependence between the treatment assignment and the imputed covariate values, thereby breaking the balance between arms created by randomization. As the result, the use of these incorrectly imputed covariates may absorb part of the effect that should have been attributed to the randomized treatment, leading to bias even in the ATE estimate.

Incompatibility in the MI of covariates is a challenge encountered in working with both randomized and non-randomized data. The general solution is to preserve the substantive model structure in the imputation model; see, for example [2, 25, 39],. However, this can be computationally challenging.

#### Multiple imputation by randomization arm

In RCTs with continuous outcomes, incompatibility may be addressed in a simple way—namely, by specifying the imputation model within each randomization arm. In our example, this involves fitting $\:{X}_{2}={X}_{1}+Y+{\epsilon\:}_{2}$ separately for the treatment and control arms when constructing the imputation model, which can be easily implemented using standard MI software.

There is an intuitive explanation to how “MI by arm” works around the incompatibility problem and correctly capture the treatment effect heterogeneity. Recall that the data-generating model is assumed to be $\:Y={\:\beta\:}_{0}+{\:\beta\:}_{1}T+{\:\beta\:}_{2}{X}_{1}+{\:\beta\:}_{3}{X}_{2}+{\:\beta\:}_{4}{X}_{1}T+{\:\beta\:}_{5}{X}_{2}T+\epsilon\:$. If so, it can alternatively be written as two separate linear models with different parameter values for the two experimental arms:

$$\:Y={\:\beta\:}_{0}{\prime\:}+{\:\beta\:}_{1}{\prime\:}{X}_{1}+{\:\beta\:}_{2}{\prime\:}{X}_{2}+\epsilon\:\:\text{f}\text{o}\text{r}\:T=1,\:\text{a}\text{n}\text{d}$$

$$\:Y={\:\beta\:}_{0}{\prime\:}{\prime\:}+{\:\beta\:}_{1}{\prime\:}{\prime\:}{X}_{1}+{\:\beta\:}_{2}{\prime\:}{\prime\:}{X}_{2}+\epsilon\:\:\text{f}\text{o}\text{r}\:T=0,$$

where

$$\:{X}_{2}={\:\gamma\:}_{0}{\prime\:}+{\:\gamma\:}_{1}{\prime\:}{X}_{1}+{\:\gamma\:}_{2}{\prime\:}Y+{\epsilon\:}_{2}\:\text{f}\text{o}\text{r}\:T=1,\:\text{a}\text{n}\text{d}$$

$$\:{X}_{2}={\:\gamma\:}_{0}{\prime\:}{\prime\:}+{\:\gamma\:}_{1}{\prime\:}{\prime\:}{X}_{1}+{\:\gamma\:}_{2}{\prime\:}{\prime\:}Y+{\epsilon\:}_{2}\:\text{f}\text{o}\text{r}\:T=0.$$.

These models could be used for imputing $\:{X}_{2}$. Within each arm, the arm-specific linear model of $\:Y$ is compatible with the corresponding linear model for $\:{X}_{2}$, even though no interaction terms are explicitly included.

Under MAR, “MI by arm” is able to produce unbiased estimates for both the ATE and subgroup treatment effects. Sullivan et al. [4] recommended MI by arm for handling missingness in the RCT *outcome;* here, we additionally point out that this method has advantages for handling missingness in the *covariates*.

However, there are three caveats related to “MI by arm”. First, the idea of using “MI by arm” to resolve the problem of incompatibility between the imputation model and substantive analysis model only works when the outcome is continuous. If the outcome is, for example, binary and modeled by logistic regression, then the two substantive models will remain nonlinear even after splitting by arm. The implied within-arm model, $\:\text{l}\text{o}\text{g}\text{i}\text{t}\left(\text{E}(\text{Y}\right))={\:\gamma\:}_{0}+{\:\gamma\:}_{1}X,$ would not provide an analytic way to solve for $\:E\left(X\right)$. Thus, for non-continuous outcomes, other approaches that accommodate the substantive analysis model in the imputation model are still needed [2, 25, 31].

Second, “MI by arm” requires a sufficient sample size in each arm. When the overall sample size is limited, splitting the data may result in higher sampling variance in each imputation model, leading to increased variance in subsequent treatment effect estimates. Therefore, the choice between “MI overall” and “MI by arm” can be seen as a bias-variance tradeoff (see 47). “MI overall” pools data from both experimental arms to generates a more stable imputation model, but it may misrepresent relationships between covariates and the outcome, which could introduce biases into the estimated treatment effects. Conversely, “MI by arm” accounts for the interactions between covariates and the treatment and can therefore estimate treatment effects with less bias; but it also leads to greater sampling variance for the estimates.

Third, the MAR assumption is required for “MI by arm” to generate unbiased treatment effects. If covariates are missing not at random (MNAR), then the two imputation models by arm may both fail to capture the true distributions of the covariates, and they may distort the true distributions in different ways that lead to imbalance in the imputed covariate values between the two arms and result in biased ATE and subgroup treatment effect estimates. We present a toy example in Appendix 1 to illustrate this kind of bias. Notably, the impact of an MNAR mechanism is particularly relevant when considering “MI by arm”, as this is the approach where unbiased ATE and subgroup treatment effects are achievable under MAR. For the other two MI approaches discussed above, the imputation models are inherently mis-specified, and they give biased estimates even under MAR, so the fact that they can also be biased under MNAR is not as surprising.

#### Limitation of MI in estimating treatment effects

The bias in the ATE estimate under MI is worth highlighting because it demonstrates potential pitfalls of MI for handling covariates in *RCTs*, where the imputation method should maintain the independence between covariates and the randomized treatment assignment. As mentioned above, one way is to use only baseline variables; although this preserves the independence, it mis-specifies the model and leads to biased estimates for any coefficients related to covariates. Alternatively, imputed covariates are balanced between arms when the imputation model is correctly specified *and* the MAR assumption holds. If the model is mis-specified, as is the case with “MI overall”, or if the MAR is violated, as happens with “MI by arm” under MNAR, then the balance achieved by randomization could be compromised, leading to bias in the ATE. Because of these potential complexities and limitations, researchers may be interested in whether a simpler alternative can do just as well or perhaps better than MI.

### Simple general strategies

RCTs are considered the gold standard for estimating treatment effects because randomization helps to balance confounders between experimental arms. Under this premise, for the purpose of estimating ATE, missingness in covariates could be handled by leveraging the randomization design. We describe two simple, general strategies, which are not explicitly based on statistical models but simply manipulate the dataset so that the originally proposed analysis can be run on the incomplete dataset without excluding cases: grand mean imputation (GMI) and the missing indicator method (MIM). We emphasize that these methods are not a general solution for missing data, but they can be useful in the RCT context for managing incomplete covariates when estimating the ATE.

The utility of these general strategies (GMI and MIM) may seem counterintuitive to investigators accustomed to working with observational study data, because single imputation is strongly discouraged by several literature reviews [19–21, 30]. Nonetheless, these strategies are appropriate for the ATE in RCTs because they use partial information of the incomplete covariates in a specific way that achieves two goals simultaneously: (1) not excluding any experimental units due to missing data in covariates; (2) retaining the expected balance in covariates between experimental arms established by the randomization. Not all data manipulation strategies achieve these two goals. For example, a complete-case analysis fails to achieve the first goal as it excludes cases with incomplete covariates. Single imputation based on a regression model typically fails to achieve the second goal because it cannot impose balance in imputed covariates between arms, unless the regression model is specified in a restricted way (e.g., intercept only, which would then be equivalent to GMI).

#### Grand mean imputation (GMI)

With GMI, all missing values of a covariate are replaced with the observed overall sample mean; the imputed covariate is then used as if it were fully observed. This approach is usually not recommended, as it distorts the estimated coefficients by attenuating the correlation between a mean-imputed covariate and the outcome toward zero [40]. With non-RCT data where variables are mutually correlated, the biased coefficient estimation of one predictor can subsequently bias the coefficients of other predictors; hence, none of the regression coefficients is ensured to be unbiased when GMI is applied to handle missingness in any predictor.

Nonetheless, in RCTs, covariates are (in expectation) independent of the treatment assignment by design, and thus, the point estimate of the treatment coefficient is not biased by (mis)specification of other covariates. Applying GMI to incomplete covariates leads to an unbiased ATE for two reasons: (1) Intention to treat is respected in that ATE is estimated on all units that are the subject of the randomization (i.e., no rows are dropped in a way that could cause imbalance). (2) By imputing a mean score to all missing values of a covariate, this approach also preserves the independence of the imputed covariates from the treatment assignment. The inferential validity here is grounded in the randomization design, and does not rely on untestable assumptions about missing data mechanism (MAR) as MI would. For an intuition into why the partial loss of covariate information is not a problem, consider that even omitting covariates from the analysis model altogether would still not have biased the ATE, because the covariates are being used only to reduce error variance, not to correct for bias. The lost information would lead to lower precision, but not to bias, because the lost information was independent of the treatment effect. Notably, it is also the reason why “baseline-only MI” discussed above can yield an unbiased estimate of ATE, despite incorrectly modeling the relationships between imputed covariates and the outcome.

Of course, imputing a grand mean for missing covariate values *does* distort the relationships between covariates and the outcome, leading to two caveats. First, the inferential validity is limited to the ATE (coefficient of $\:T$) and does not apply to effects related to covariates, such as subgroup treatment effects (e.g., coefficient of $\:{X}_{1}T$ and $\:{X}_{2}T$) or coefficient of covariates (e.g., coefficient of $\:{X}_{1}$ and $\:{X}_{2}$). Second, like all missing data approaches, GMI is unable to restore the information which would have been present if the covariate had been fully observed. Mean-imputed covariates explain less of the outcome variance and contribute less to improving the precision of estimates than they would if fully observed. However, this approach is still preferable to excluding incomplete covariates altogether. In our example, when the GMI is applied to handle incomplete covariates, the two substantive models would be fitted as:

Primary Model: $$\begin{aligned}\:Y&={\beta\:}_{0}+{\:\beta\:}_{1}T+{{\:\beta\:}_{2}X}_{1}\\&+{\:\beta\:}_{3}{{X}_{2}}^{GMI}+\epsilon\:\end{aligned}$$

Secondary Model: $$\begin{aligned}\:Y&={{\beta\:}_{0}+\:\beta\:}_{1}T+{\:\beta\:}_{2}{X}_{1}\\&+{\beta\:}_{3}{{X}_{2}}^{GMI}+{\beta\:}_{4}{X}_{1}T\\&+{\:\beta\:}_{5}{{X}_{2}}^{GMI}T+\epsilon\:\end{aligned}$$,

where $\:{{X}_{2}}^{GMI}$ indicates $\:{X}_{2}\:$when missing values are grand mean imputed from the observed values of $\:{X}_{2}$.

#### Missing indicator method (MIM)

MIM is another strategy that has been criticized for introducing biases into coefficient estimates in observational studies [20, 21, 30] but that can be applied to estimate the ATE in RCTs without bias [15–18]. Under this approach, missing values in covariates are imputed with a single constant, where the choice of the constant value only influences the intercept term. Then, an additional missingness indicator (1 = missing, 0 = observed) is created and used with the singly imputed covariate as a pair.

MIM can be used for handling incomplete covariates in RCTs to produce an unbiased estimate of the ATE for a similar reason as GMI. Assuming random assignment, both the imputed covariate and the missingness indicator are orthogonal to the treatment assignment in a large sample sense, just as the complete covariate would have been. The point estimate of the ATE is asymptotically independent of covariate adjustment. Again, the MIM is unbiased because of the randomization procedure’s property of balancing confounders, and it does not require assumptions about missing data mechanisms. However, in the same way as GMI, when the MIM is used on covariates, the regression coefficient related to covariates could be biased and should not be interpreted. Under the MIM, the two substantive models in our example would be fitted as:

Primary Model: $$\begin{aligned}\:Y&={\beta\:}_{0}+{\:\beta\:}_{1}T+{\beta\:}_{2}{X}_{1}\\&+{\beta\:}_{3}{{X}_{2}}^{MIM}+{\beta\:}_{4}{M}_{2}+\epsilon\:\end{aligned}$$

Secondary Model: $$\begin{aligned}\:Y&={\beta\:}_{0}+{\:\beta\:}_{1}T+{\beta\:}_{2}{X}_{1}+{\beta\:}_{3}{{X}_{2}}^{MIM}\\&+{\beta\:}_{4}{M}_{2}+{\beta\:}_{5}{X}_{1}T+{\beta\:}_{6}{{X}_{2}}^{MIM}T\\&+{\beta\:}_{7}{M}_{2}T+\epsilon\:\end{aligned}$$,

where a missingness indicator $\:{M}_{2}$ is included in addition to the singly imputed $\:{{X}_{2}}^{MIM}$.

#### Grand mean imputation versus missing indicator method as a Bias-Variance tradeoff

GMI and MIM both provide unbiased estimates of the ATE (but biased subgroup treatment effects), but they may differ in statistical efficiency depending on the sample size and nature of missingness. The MIM can be more efficient than GMI when the probability of missingness is related to the experimental outcome because, in this case, missingness indicators represent important covariates that can explain additional variance of the outcome. However, if the sample size is modest or the list of covariates is long, posing a concern of overfitting, then GMI corresponds to a more parsimonious model and may produce a more precise ATE than the MIM [17].

## Simulation results

We conducted a simulation study to illustrate the advantages and disadvantages discussed above for different strategies for the ATE and subgroup treatment effects. We considered both MAR and MNAR, paired with scenarios that represent different data-generating mechanisms ($\:{M}_{2}$ directly correlated vs. not correlated with $\:Y$; heterogenous vs. homogeneous treatment effects), different amounts of missingness (50% vs. 25%), different sample sizes (1000 vs. 100), and different types of covariates (continuous vs. binary). Without loss of generality, we present results under one setting in the main text (i.e., $\:{M}_{2}$ not directly correlated with $\:Y$, heterogeneous treatment effect, 50% missing rate, 1000 sample size, and continuous covariates); full details are reported in Appendix 2.

Building on the setup described above in Sect. 2, we randomly assign 50% of simulated cases to each arm. A bivariate normal distribution is specified for the two covariates: $$\:\begin{bmatrix}X_1\\\:X_2\end{bmatrix}\sim N\begin{pmatrix}\begin{bmatrix}0\\\:0\end{bmatrix}&\:\begin{bmatrix}1&\:0.3\\\:0.3&\:1\end{bmatrix}\end{pmatrix}$$, where $\:{X}_{1}$ is fully observed and $\:{X}_{2}$ is partly missing. $\:{M}_{2}$ is a binary variable indicating whether $\:{X}_{2}$ is missing [41] or observed (0). (In Appendix 2, we considered an alternative scenario where $\:{X}_{1}$ and $\:{X}_{2}$ are binary variables.)

We considered two missing data mechanisms: *Under MAR*, the probability that $\:{X}_{2}$ will be missing is a function of $\:{X}_{1}$: specifically, $\:{logit\left(P\right(M}_{2}=1\left)\right)=3{X}_{1}$. *Under MNAR*, the probability of missing data of $\:{X}_{2}$ is set to be a function of both $\:{X}_{1}$ and the true value of $\:{X}_{2}$: $\:{\text{l}\text{o}\text{g}\text{i}\text{t}\left(P\right(M}_{2}=1\left)\right)=3{X}_{1}+3{X}_{2}$. For both MAR and MNAR, the setup results in about 50% missing for $\:{X}_{2}$. (In Appendix 2, we considered an alternative scenario with about 25% missing rate.)

The potential outcomes $\:Y\left(T\right)$ are generated as

$$\:Y\left(T\right)=1+{X}_{1}+{X}_{2}+2T+2{X}_{1}T+{2X}_{2}T+e$$,

where $\:e\sim N\left(\text{0,1}\right)$ indicates the random error. (In Appendix 2, we considered an alternative data-generating model where $\:Y$ is a function of $\:{M}_{2}$ and the result of a homogenous treatment effect.) We generated 5000 replicate samples; each contained $\:n=1000$ participants. (In Appendix 2, we considered an alternative scenario with a smaller sample size $\:n=100.$) In each replicate sample, we implemented five missing data strategies—1) MI with only baseline variables, 2) MI overall, 3) MI by arm, 4) GMI, and 5) MIM—along with 6) a complete-case analysis (i.e., excluding participants with missing data in covariates from the analysis) and 7) an unadjusted ATE estimate for comparison. With each strategy, we fitted two substantive analysis models. We study the coefficient of $\:T$ in the primary analysis model and coefficients of $\:T$, $\:{X}_{1}T$, and $\:{X}_{2}T$ in the secondary analysis model. We centered covariates at their sample mean values in all analyses so that the coefficient of $\:T$ represents the ATE in both models.

We report the average point estimate, standard error (SE), mean squared error (MSE), and coverage rate of 95% confidence intervals based on the results pooled across replicate samples. For the three MI strategies, we created $\:m=20$ imputed datasets for each simulated dataset; 95% CIs were constructed based on the *t* distribution with 19 degrees of freedom (i.e., $\:m-1$). For all other strategies, 95% CIs were constructed based on a normal distribution. The simulation is performed in R 4.4.2 [42]; we used the package *mice* [38] for MI. Tables 1 and 2 report the primary analysis (coefficient of $\:T$) and secondary analysis (coefficients of $\:T$, $\:T$, $\:{X}_{1}T$, and $\:{X}_{2}T$) results under the MAR mechanism; Tables 3 and 4 report the primary and secondary analysis results under the MNAR mechanism.

**Table 1:** Mean estimated coefficients of $\:T\:$and standard errors in the Primary-Analysis model under MAR (covariates are mean-centered)

	Average Estimate	SE	MSE	95% CI coverage
Target estimand (true value)	2.000			
Unadjusted^1^	2.000	0.238	0.056	94.8%
Complete case	0.210	0.150	3.225	0.0%
MI baseline-only	1.999	0.156	0.025	96.9%
MI overall	0.990	0.158	1.045	0.0%
MI by arm	1.990	0.169	0.029	95.4%
Grand mean	2.001	0.151	0.023	94.7%
Missing indicator	2.001	0.151	0.023	94.6%

^1^ The unadjusted ATE is presented here for the comparison purpose and estimated using model$\:Y\sim T$with no covariates.

**Table 2:** Mean estimated coefficients of $\:T$, $\:{X}_{1}T$, and $\:{X}_{2}T$ and standard errors in the Secondary-Analysis model under MAR (covariates are mean-centered)

	Average Estimate	SE	MSE	95% CI coverage
$$\:T$$				
Target estimand (true value)	2.000			
Complete case	1.586	0.171	0.201	16.7%
MI baseline-only	1.999	0.156	0.024	94.0%
MI overall	1.120	0.146	0.796	0.0%
MI by arm	1.994	0.169	0.029	88.2%
Grand mean	1.999	0.151	0.023	86.7%
Missing indicator	1.999	0.151	0.023	86.5%
$$\:{X}_{1}T$$				
Target estimand (true value)	2.0000			
Complete case	2.000	0.127	0.016	95.1%
MI baseline-only	2.340	0.172	0.145	46.0%
MI overall	1.214	0.106	0.629	0.0%
MI by arm	2.001	0.113	0.013	95.4%
Grand mean	2.450	0.123	0.218	3.4%
Missing indicator	2.311	0.168	0.125	52.8%
$$\:{X}_{2}T$$				
Target estimand (true value)	2.000			
Complete case	2.002	0.095	0.009	94.9%
MI baseline-only	1.034	0.154	0.956	0.0%
MI overall	1.775	0.102	0.061	57.0%
MI by arm	2.038	0.095	0.010	91.5%
Grand mean	1.927	0.100	0.015	99.6%
Missing indicator	1.950	0.099	0.012	99.8%

**Table 3:** Mean estimated coefficients of $\:T\:$and standard errors in the Primary-Analysis model under MNAR (covariates are mean-centered)

	Average Estimate	SE	MSE	95% CI coverage
Target estimand (true value)	2.000			
Unadjusted^1^	2.002	0.237	0.056	94.9%
Complete case	−0.409	0.133	5.822	0.0%
MI baseline-only	2.003	0.159	0.025	97.0%
MI overall	0.604	0.180	1.981	0.0%
MI by arm	1.348	0.174	0.455	4.4%
Grand mean	2.005	0.153	0.024	95.1%
Missing indicator	2.004	0.141	0.020	95.3%

^1^ The unadjusted ATE is presented here for the comparison purpose and estimated using model$\:Y\sim T$with no covariates.

**Table 4:** Mean estimated coefficients of $\:T$, $\:{X}_{1}T$, and $\:{X}_{2}T$ and standard errors in the Secondary-Analysis model under MNAR (covariates are mean-centered)

	Average Estimate	SE	MSE	95% CI coverage
$$\:T$$				
Target estimand (true value)	2.000			
Complete case	0.794	0.148	1.477	0.0%
MI baseline-only	2.002	0.158	0.025	94.0%
MI overall	0.839	0.158	1.373	0.0%
MI by arm	1.403	0.156	0.381	2.2%
Grand mean	2.002	0.153	0.024	89.0%
Missing indicator	2.001	0.140	0.020	84.6%
$$\:{X}_{1}T$$				
Target estimand (true value)	2.000			
Complete case	1.998	0.115	0.013	95.1%
MI baseline-only	2.784	0.143	0.634	0.1%
MI overall	1.490	0.127	0.276	2.7%
MI by arm	1.958	0.108	0.013	93.4%
Grand mean	2.666	0.122	0.458	0.0%
Missing indicator	1.897	0.134	0.029	86.6%
$$\:{X}_{2}T$$				
Target estimand (true value)	2.000			
Complete case	2.003	0.113	0.013	95.8%
MI baseline-only	1.091	0.170	0.855	1.9%
MI overall	1.930	0.156	0.029	91.1%
MI by arm	2.208	0.139	0.063	54.0%
Grand mean	2.069	0.153	0.028	99.3%
Missing indicator	1.992	0.113	0.013	99.8%

## Simulation discussion

Before addressing missing covariates, we first highlight that the simulation results demonstrate the benefit of covariate adjustment. As reported in Tables 1 and 3, the unadjusted ATE remains unbiased but has a large standard error. This shows that covariate adjustment is useful for improving precision.

Bias in treatment effects due to missing covariates can be seen in the results of complete-case analysis. In Tables 1 and 3, the ATE estimated from complete-case analysis is highly biased. In Tables 2 and 4, while coefficients of $\:{X}_{1}T$, and $\:{X}_{2}T$ are unbiased (which is expected in our setup of the data generation and missing data mechanisms), the coefficients of $\:T$ are still highly biased, resulting in biased subgroup treatment effects (recall that coefficients of $\:T$, $\:{X}_{1}T$, and $\:{X}_{2}T$ are needed to fully specify the treatment effect for any given subgroup). These results highlight that missingness in covariates should be dealt with using a strategy other than excluding cases to avoid biased inferences.

The simulation results illustrate the properties of the missing data strategies discussed above. Interpretation of the different metrics—point estimate, SE, MSE, and 95% CI coverage rate— leads to the same conclusions. First, for the ATE (coefficient of $\:T$), baseline-only MI, GMI, and MIM produce unbiased estimates with similarly low MSE and correct CI coverage rates. This is the case regardless of whether the missing data mechanism is MAR (Table 1) or MNAR (Table 3). The results illustrate that under successful randomization, the management of missingness in covariates does not need to rely on correctly modeling the distribution of covariates and on untestable assumptions about the missing data mechanism. Instead, these strategies’ ability to produce unbiased ATE could be attributed to the balanced covariate distributions established by the randomization process.

Second, for subgroup treatment effects, “MI by arm” is the only strategy tested here that could produce generally unbiased estimates; but this strategy heavily relies on the MAR assumption. If MAR holds, then “MI by arm” is the best strategy as it simultaneously addresses the need for estimating the ATE (Table 1; coefficient of $\:T$ is 1.990) *and* subgroup treatment effects (Table 2; coefficients of $\:T$, $\:{X}_{1}T$, and $\:{X}_{2}T$ are 1.994, 2.001, and 2.038). However, if MAR is violated, then the ATE estimate could be severely biased under “MI by arm” (Table 3; coefficient of $\:T$ is 1.348). Since investigators cannot verify whether the mechanism is MAR or MNAR, methods that capitalize on the randomization design (re-randomization MI, GMI, and MIM) are potentially more attractive for estimating the ATE, because these methods provide unbiasedness without relying on assumptions about the missing data mechanism.

Third, “MI overall” produces biased estimates for the ATE (Tables 1 and 3) and subgroup treatment effects (Tables 2 and 4) under both MAR and MNAR, when the true treatment effect is assumed to be heterogeneous in the target population—an assumption that we consider necessary for real-world applications. The issue lies in the incompatibility between the imputation model of $\:{X}_{2}$ and the true data-generating model of $\:Y$. The incompatible imputation cannot ensure the imputed $\:{X}_{2}$ values to be balanced between arms, which leads to bias even in the ATE. Note that “MI overall” is the default imputation model in some standard MI software (e.g., mice); analysts working with RCT data should carefully consider that this entails the possibility of imputing values that violate the randomization design.

## Conclusion

In Table 5, we summarize the advantages and disadvantages of the different missing data strategies with respect to the two common target estimands in RCTs. We discussed that the choice of a missing data strategy depends on the target estimands and involves trade-offs. “MI by arm” may be used to estimate both the ATE and subgroup treatment effects, but its performance depends on the MAR assumption. For estimating the ATE (which may be the primary aim of the trial), GMI, MIM, and baseline-only MI all provide unbiased estimates under the assumption of successful randomization. Sensitivity analyses with different strategies for incomplete covariates can be used to assess the plausibility of respective assumptions; this may be especially important when there is a large amount of missingness in covariates.

**Table 5:** Summary of the pros and cons of the different missing data strategies with respect to the target estimand

	Average treatment effect (Primary-Analysis Aim)		Subgroup treatment effects (Secondary-Analysis Aim)	
	Pros	Cons	Pros	Cons
Unadjusted ATE	Unbiased	Not taking advantage of the variance-reduction benefit of covariate adjustment	--	--
Complete-case analysis	Unbiased (under a restrictive assumption that the true treatment effect is homogeneous)	Generally biased (under a more realistic assumption that the true treatment effect is heterogeneous)	--	Biased
MI baseline-only	Unbiased; does not depend on assumptions about missing data mechanism	Slightly more complex to implement than GMI and MIM	--	Biased
MI overall	Unbiased (under a restrictive assumption that the true treatment effect is homogeneous)	Generally biased (under a more realistic assumption that the true treatment effect is heterogeneous)	--	Biased (under a more realistic assumption that the true treatment effect is heterogeneous)
MI by arm	Unbiased (under a more realistic assumption that the true treatment effect is heterogeneous)	Depends on MAR assumption and requires sufficient sample size in each experimental arm	Unbiased	Depends on MAR assumption and requires sufficient sample size in each experimental arm
Grand mean imputation	Unbiased; Does not depend on assumptions about missing data mechanism	May underfit (may be less efficient than the missing indicator method if missingness is correlated with the outcome)	--	Biased
Missing indicator method	Unbiased; Does not depend on assumptions about missing data mechanism	May overfit (may be less efficient than the grand mean imputation if sample size is small)	--	Biased

This work has limitations but suggests interesting future directions. We have focused on clarifying the assumptions and mechanisms by which different strategies can be used to handle missing data in covariates. These strategies can readily be adapted to combine with guidelines for handling missingness in outcomes. For example, Sullivan et al. [4] suggested using “MI by arm” to address missingness in the outcome, and we have highlighted the benefit of this approach for managing missing data in covariates. Alternatively, if one does not want to risk imputing unbalanced values into covariates, one may consider using strategy like GMI to handle missingness in covariates, while still using a model-based approach (MI) for handling missingness in the outcome.

We focused on a continuous outcome with a linear model. Method performance may differ for binary or time-to-event outcomes [31, 36]. For example, when the outcome is binary and the substantive outcome is fitted as logistic regression, “MI by arm” does not circumvent the incompatibility between the (non-linear) outcome model and the (linear) imputation model. Our suggestions here should not be applied to binary outcomes (e.g., 38). Future research is needed to examine the best way to perform MI for incomplete covariates in an RCT when the experimental outcome is not continuous.

## Data Availability

The dataset generated and analyzed during the current study is available in https://github.com/ShiyuZhangGH/open-code/tree/main/missingCovariate_RCT_code.

## Appendix 1

This is a toy example illustrating the bias in the average treatment effect under “MI by arm” when $\:{X}_{2}$ is MNAR. Suppose that the true model of the experimental outcome is

$$\:Y\left(T\right)\:\sim\:1+{X}_{1}+{X}_{2}+T+{X}_{1}T+{X}_{2}T$$

.

By treating the two experimental arms as separate samples, this model can be alternatively written as

$$\:Y=\left\{\begin{array}{c}{{{\beta\:}_{1}+\beta\:}_{2}X}_{1}+{{\beta\:}_{3}X}_{2},\:\:\:T=1\\\:{{{\beta\:}_{a}+\beta\:}_{b}X}_{1}+{{\beta\:}_{c}X}_{2},\:\:\:T=0\end{array}\right.$$

.

By rearranging the terms, the imputation model of $\:{X}_{2}$ is given by

$$\:{X}_{2}=\left\{\begin{array}{c}-\frac{{\beta\:}_{1}}{{\beta\:}_{3}}+\frac{1}{{\beta\:}_{3}}Y-\frac{{\beta\:}_{2}}{{\beta\:}_{3}}{X}_{1},\:\:\:T=1\\\:-\frac{{\beta\:}_{a}}{{\beta\:}_{c}}+\frac{1}{{\beta\:}_{c}}Y-\frac{{\beta\:}_{b}}{{\beta\:}_{c}}{X}_{1},\:\:\:T=0\end{array}\right.$$

.

Thus, “MI by arm” is compatible with the true substantive model of $\:Y$.

The missing at random (MAR) assumption is the formal assumption that allows the imputation model to be estimated on the observed data as if data were not missing. However, if the mechanism is missing not at random (MNAR), meaning that the probability of missingness in $\:{X}_{2}$ depends on $\:{X}_{2}$ own values, then the parameters $\:-\frac{{\beta\:}_{1}}{{\beta\:}_{3}},\frac{1}{{\beta\:}_{3}}$, $\:-\frac{{\beta\:}_{2}}{{\beta\:}_{3}},\:\frac{{\beta\:}_{a}}{{\beta\:}_{c}},\frac{1}{{\beta\:}_{c}},-\frac{{\beta\:}_{b}}{{\beta\:}_{c}}$ cannot be estimated accurately based on the observed data. A consequence of failing to recover the missing values of $\:{X}_{2}$ is that imputed $\:{X}_{2}$ may not be balanced between the two experimental arms. For example, the imputed $\:{X}_{2}$ in the control arm ($\:T=0$) may on average have lower values than the imputed $\:{X}_{2}$ in the experimental arm ($\:T=1$). In that case, the imputation of a covariate will undermine the independence between the covariate and the randomization assignment established by the randomization procedure, and in turn leads to a biased estimate of the average treatment effect.

## Appendix 2

### Summary of simulation scenarios

Our simulation study evaluated the performance of different missing data strategies for managing missing covariates when estimating the ATE and subgroup treatment effects. We considered both MAR and MNAR, paired with scenarios that represent different data-generating mechanisms ($\:{M}_{2}$ correlated vs. not correlated with $\:Y$; heterogenous vs. homogeneous treatment effects; heterogenous vs. homogeneous treatment effects), different amounts of missingness (50% vs. 25%), different sample sizes (1000 vs. 100), and different types of covariates (continuous vs. binary). The main text presents the results under one combination of scenario settings. In this appendix, we report the results under four additional scenarios, each of which varies one setting from the main text scenario while holding the others constant. The scenario settings reported in the main text and different sections of this appendix are summarized in Table a in Appendix 2.

**Taba:** Simulation scenarios reported in the main text versus different sections of this appendix

		Main Text	Section B	Section C	Section D	Section E
Data-generating mechanism	$\:{M}_{2}$ not correlated with $\:Y$	✓		✓	✓	✓
	$\:{M}_{2}$ correlated with $\:Y$		✓			
$\:{M}_{2}$ – $\:Y$ correlation	No	✓		✓	✓	✓
	Yes		✓			
Missing rate	50%	✓	✓		✓	✓
	25%			✓		
Sample size	1000	✓	✓	✓		✓
	100				✓	
Covariate type	Continuous	✓	✓	✓	✓	
	Binary					✓

### Different data-generating model

In the main text, we assumed the true data-generating model is $\:Y\left(T\right)=1+{X}_{1}+{X}_{2}+2T+2{X}_{1}T+{2X}_{2}T+e$, which has two noticeable properties. First, the treatment effect is heterogeneous depending on the values of $\:{X}_{1}$ and $\:{X}_{2}$; this is a complexity that we considered necessary in real-world applications. Second, missingness ($\:{M}_{2}$) is not prognostic of the outcome. In this Appendix, we explore an alternative data-generating model:

$$\:Y\left(T\right)=1+{X}_{1}+{X}_{2}+{M}_{2}+2T.$$

Here, the outcome is directly related to $\:{M}_{2}$ and is the result of a homogeneous treatment effect. All other settings of the simulation are the same as discussed in the main text. The primary analysis results under MAR and MNAR are reported in Tables b and c in Appendix 2, respectively. The results are consistent with what would be expected from the theoretical properties of the missing data strategies. (1) “MI overall” generates unbiased ATE under MAR, because the imputation model used in “MI overall” is now compatible with the true outcome model. (2) MIM is more efficient than GMI because MIM includes the missing indicator ($\:{M}_{2}$) as a covariate, which explains additional error variance in the outcome.

**Tabb:** Mean estimated coefficients of $\:T\:$and standard errors in the Primary-Analysis model under MAR (under an alternative data-generating model)

	Average Estimate	SE	MSE	95% CI coverage
Unadjusted^1^	2.000	0.137	0.019	95.5%
Complete case	2.000	0.089	0.008	95.0%
MI baseline-only	2.000	0.081	0.007	97.5%
MI overall	2.000	0.078	0.006	96.5%
MI by arm	1.999	0.107	0.012	93.2%
Grand mean	1.999	0.080	0.006	95.2%
Missing indicator	2.000	0.076	0.006	95.1%

^1^ The unadjusted ATE is presented here for the comparison purpose; by definition, it is estimated using model$\:Y\sim T$with no covariates.

**Tabc:** Mean estimated coefficients of $\:T$ and standard errors in the Primary-Analysis model under MNAR (under an alternative data-generating model)

	Average Estimate	SE	MSE	95% CI coverage
Unadjusted^1^	1.999	0.142	0.020	95.3%
Complete case	2.000	0.090	0.008	95.1%
MI baseline-only	2.000	0.094	0.009	97.2%
MI overall	1.998	0.085	0.007	96.6%
MI by arm	1.998	0.124	0.015	92.6%
Grand mean	1.999	0.091	0.008	95.5%
Missing indicator	1.999	0.071	0.005	95.4%

^1^ The unadjusted ATE is presented here for the comparison purpose; by definition, it is estimated using model$\:Y\sim T$with no covariates.

### Lower missingness rate

The main text reports results based on approximately 50% missing data in covariate $\:{X}_{2}$. Here, we present results for an alternative scenario with a lower missingness rate of about 25%. Specifically, we specify the MAR missing data mechanism for $\:{X}_{2}$ as $\:{logit\left(P\right(M}_{2}=1\left)\right)=-2.5+3{X}_{1}$ and the MNAR missing data mechanism as $\:{\text{l}\text{o}\text{g}\text{i}\text{t}\left(P\right(M}_{2}=1\left)\right)=-2.5+3{X}_{1}+3{X}_{2}$. The primary and secondary analysis results under MAR are reported in Tables d and e in Appendix 2; the primary and secondary analysis results under MNAR are reported in Tables f and g in Appendix 2.

Overall, with less missingness, the bias is expected to be less pronounced regardless of the missing data strategy used. This can be seen, for example, by comparing Table f in Appendix 2 with Table 1 in the main text. Under complete-case analysis, the estimated ATE is 0.210 when 50% data are missing in $\:{X}_{2}$ and 1.099 when 25% data are missing (with the true value being 2). Although the magnitude of bias differs, the overall findings are consistent with those reported in the main text, as well as with the conceptual discussion: “MI by arm” is the strategy that generates unbiased estimates of both ATE and subgroup treatment effects under MAR. Capitalizing on the randomization design, “baseline-only MI”, GMI, and MIM generate unbiased ATE regardless of the missing data mechanism, but they cannot be used for estimating subgroup treatment effects.

**Tabd:** Mean estimated coefficients of $\:T\:$and standard errors in the Primary-Analysis model under MAR (missing rate around 25%)

	Average Estimate	SE	MSE	95% CI coverage
Unadjusted^1^	2.002	0.233	0.054	95.2%
Complete case	1.099	0.127	0.827	0.0%
MI baseline-only	2.001	0.138	0.019	97.9%
MI overall	1.510	0.126	0.256	3.8%
MI by arm	1.999	0.132	0.017	96.1%
Grand mean	2.001	0.137	0.019	94.9%
Missing indicator	2.001	0.136	0.018	95.1%

^1^ The unadjusted ATE is presented here for the comparison purpose; by definition, it is estimated using model$\:Y\sim T$with no covariates.

**Tabe:** Mean estimated coefficients of $\:T$, $\:{X}_{1}T$, and $\:{X}_{2}T$ and standard errors in the Secondary-Analysis model under MAR (missing rate around 25%)

	Average Estimate	SE	MSE	95% CI coverage
$$\:T$$				
Complete case	1.793	0.130	0.060	34.9%
MI baseline-only	2.001	0.138	0.019	92.0%
MI overall	1.555	0.124	0.213	2.0%
MI by arm	2.000	0.132	0.017	79.4%
Grand mean	2.000	0.137	0.019	81.7%
Missing indicator	2.001	0.136	0.018	81.2%
$$\:{X}_{1}T$$				
Complete case	2.000	0.091	0.008	95.1%
MI baseline-only	2.169	0.148	0.050	77.7%
MI overall	1.442	0.086	0.318	0.0%
MI by arm	2.003	0.086	0.007	95.6%
Grand mean	2.306	0.117	0.108	15.3%
Missing indicator	2.109	0.117	0.026	86.4%
$$\:{X}_{2}T$$				
Complete case	2.000	0.078	0.006	94.7%
MI baseline-only	1.540	0.119	0.226	11.8%
MI overall	1.880	0.081	0.021	84.3%
MI by arm	2.011	0.077	0.006	95.3%
Grand mean	1.937	0.082	0.011	97.1%
Missing indicator	1.977	0.079	0.007	99.2%

**Tabf:** Mean estimated coefficients of $\:T$ and standard errors in the Primary-Analysis model under MNAR (missing rate around 25%)

	Average Estimate	SE	MSE	95% CI coverage
Unadjusted^1^	2.000	0.240	0.057	94.7%
Complete case	0.438	0.120	2.455	0.0%
MI baseline-only	2.000	0.155	0.024	97.1%
MI overall	1.124	0.141	0.787	0.0%
MI by arm	1.632	0.136	0.154	26.1%
Grand mean	2.001	0.148	0.022	94.6%
Missing indicator	2.000	0.134	0.018	94.9%

^1^ The unadjusted ATE is presented here for the comparison purpose; by definition, it is estimated using model$\:Y\sim T$with no covariates.

**Tabg:** Mean estimated coefficients of $\:T$, $\:{X}_{1}T$, and $\:{X}_{2}T$ and standard errors in the Secondary-Analysis model under MNAR (missing rate around 25%)

	Average Estimate	SE	MSE	95% CI coverage
$$\:T$$				
Complete case	1.217	0.125	0.629	0.0%
MI baseline-only	1.999	0.154	0.024	92.5%
MI overall	1.252	0.133	0.578	0.0%
MI by arm	1.657	0.132	0.135	12.5%
Grand mean	2.000	0.148	0.022	85.3%
Missing indicator	1.999	0.133	0.018	79.4%
$$\:{X}_{1}T$$				
Complete case	1.999	0.092	0.009	94.9%
MI baseline-only	2.703	0.128	0.511	0.1%
MI overall	1.555	0.102	0.208	1.6%
MI by arm	1.961	0.089	0.009	94.2%
Grand mean	2.597	0.112	0.369	0.0%
Missing indicator	1.891	0.109	0.024	82.0%
$$\:{X}_{2}T$$				
Complete case	2.000	0.091	0.008	94.8%
MI baseline-only	1.331	0.187	0.483	19.2%
MI overall	1.988	0.112	0.013	96.3%
MI by arm	2.118	0.102	0.024	74.4%
Grand mean	1.997	0.115	0.013	99.4%
Missing indicator	2.001	0.092	0.008	99.4%

### Smaller sample size

The results reported in the main text are based on sample size $\:n=1000$ participants. Here, we consider an alternative scenario with a small sample size $\:n=100$. With a smaller sample size, the efficiency of “MI by arm” may be reduced because splitting the data by arm may result in higher sampling variance in each imputation model.

However, in our current setting, “MI by arm” performed well in simulations with $\:n=100$. Across 5000 replicate samples, all imputation models converged. “MI by arm” remains the only strategy tested there that generates unbiased ATE and subgroup treatment effect under MAR. The primary and secondary analysis results under MAR are reported in Tables h and i in Appendix 2; the primary and secondary analysis results under MNAR are reported in Tables j and k in Appendix 2.

It is worth noting that in our setting, bias (rather than variance) is the main driving factor of MSE. We do not observe a bias-variance trade-off between “MI by arm” (unbiased but has higher variance) and “MI overall” (biased but has lower variance). In other scenarios where bias is less substantial (e.g., due to lower amounts of missing data, homogeneous treatment effects etc.), or where there were many more covariates, “MI overall” might yield a smaller MSE.

**Tabh:** Mean estimated coefficients of $\:T$ and standard Errors in the Primary-Analysis model under MAR (small sample size)

	Average Estimate	SE	MSE	95% CI coverage
Unadjusted^1^	1.989	0.759	0.576	93.9%
Complete case	0.214	0.498	3.438	4.9%
MI baseline-only	2.000	0.499	0.249	96.7%
MI overall	1.226	0.514	0.863	67.5%
MI by arm	1.937	0.525	0.280	95.3%
Grand mean	2.016	0.487	0.237	93.8%
Missing indicator	2.018	0.487	0.238	93.7%

^1^ The unadjusted ATE is presented here for the comparison purpose; by definition, it is estimated using model$\:Y\sim T$with no covariates.

**Tabi:** Mean estimated coefficients of $\:T$, $\:{X}_{1}T$, and $\:{X}_{2}T$ and standard errors in the Secondary-Analysis model under MAR (small sample size)

	Average Estimate	SE	MSE	95% CI coverage
$$\:T$$				
Complete case	1.574	0.548	0.481	74.7%
MI baseline-only	1.993	0.492	0.242	93.8%
MI overall	1.313	0.474	0.698	61.5%
MI by arm	1.963	0.519	0.271	87.9%
Grand mean	1.996	0.475	0.226	87.0%
Missing indicator	1.996	0.474	0.224	87.0%
$$\:{X}_{1}T$$				
Complete case	1.998	0.452	0.205	94.0%
MI baseline-only	2.404	0.441	0.358	91.4%
MI overall	1.546	0.382	0.351	81.2%
MI by arm	2.069	0.370	0.141	95.9%
Grand mean	2.457	0.402	0.371	77.4%
Missing indicator	2.313	0.556	0.407	90.5%
$$\:{X}_{2}T$$				
Complete case	1.994	0.329	0.108	94.8%
MI baseline-only	1.081	0.393	0.999	84.0%
MI overall	1.676	0.359	0.234	94.1%
MI by arm	2.146	0.356	0.148	95.6%
Grand mean	1.917	0.350	0.129	99.7%
Missing indicator	1.942	0.347	0.124	99.8%

**Tabj:** Mean estimated coefficients of $\:T\:$and standard errors in the Primary-Analysis model under MNAR (small sample size)

	Average Estimate	SE	MSE	95% CI coverage
Unadjusted^1^	2.010	0.764	0.584	94.0%
Complete case	-0.386	0.443	5.889	0.0%
MI baseline-only	2.018	0.515	0.266	96.4%
MI overall	1.031	0.543	1.234	52.2%
MI by arm	1.534	0.550	0.520	84.9%
Grand mean	2.039	0.504	0.255	94.1%
Missing indicator	2.039	0.461	0.214	94.0%

^1^ The unadjusted ATE is presented here for the comparison purpose; by definition, it is estimated using model$\:Y\sim T$with no covariates.

**Tabk:** Mean estimated coefficients of $\:T$, $\:{X}_{1}T$, and $\:{X}_{2}T$ and standard errors in the Secondary-Analysis model under MNAR (small sample size)

	Average Estimate	SE	MSE	95% CI coverage
$$\:T$$				
Complete case	0.800	0.484	1.675	18.9%
MI baseline-only	2.010	0.511	0.261	93.2%
MI overall	1.164	0.501	0.949	48.8%
MI by arm	1.579	0.520	0.447	75.0%
Grand mean	2.016	0.497	0.247	88.1%
Missing indicator	2.006	0.447	0.200	85.4%
$$\:{X}_{1}T$$				
Complete case	1.998	0.403	0.163	94.2%
MI baseline-only	2.724	0.435	0.713	75.8%
MI overall	1.815	0.389	0.185	93.4%
MI by arm	2.158	0.360	0.154	95.5%
Grand mean	2.680	0.408	0.628	62.1%
Missing indicator	1.898	0.447	0.210	93.3%
$$\:{X}_{1}T$$				
Complete case	1.998	0.409	0.167	94.1%
MI baseline-only	1.096	0.489	1.057	93.1%
MI overall	1.900	0.458	0.220	98.1%
MI by arm	2.384	0.490	0.388	91.0%
Grand mean	2.060	0.569	0.327	99.1%
Missing indicator	1.988	0.409	0.167	99.6%

### Binary covariates

The results reported in the main text are based on continuous covariates. Subgroup analysis in RCTs often deals with binary or categorical covariates. Here, we consider an alternative scenario where $\:{X}_{1}$ and $\:{X}_{2}$ are binary variables and are positively correlated. Specifically, we first defined the continuous latent variables underlying $\:{X}_{1}$ and $\:{X}_{2}$ jointly distributed with a bivariate normal distribution $$\:N\left(\begin{array}{cc}\left[\begin{array}{c}0\\\:0\end{array}\right]&\:\left[\begin{array}{cc}1&\:0.3\\\:0.3&\:1\end{array}\right]\end{array}\right)$$. We then dichotomize the two latent variables: values greater than or equal to 0 are coded as 1; values lower than 0 are coded as -1 for both $\:{X}_{1}$ and $\:{X}_{2}$. All other settings are the same as those presented in the main text. The primary and secondary analysis results under MAR are reported in Tables l and m in Appendix 2; the primary and secondary analysis results under MNAR are reported in Tables n and o in Appendix 2.

We expect that the findings observed with continuous covariates would generalize to binary covariates. This is consistent with the simulation results: Under MAR, both the ATE and subgroup treatment effects are unbiased using “MI by arm”. Regardless of the missing data mechanism, the ATE (but not subgroup treatment effect) estimate is unbiased using “baseline-only MI”, GMI, or MIM.

**Tabl:** Mean estimated coefficients of $\:T$ and standard errors in the Primary-Analysis model under MAR (binary covariates)

	Average Estimate	SE	MSE	95% CI coverage
Unadjusted^1^	1.998	0.228	0.052	95.0%
Complete case	-0.171	0.133	4.731	0.0%
MI baseline-only	2.000	0.151	0.023	97.5%
MI overall	0.891	0.194	1.268	0.1%
MI by arm	1.989	0.223	0.050	95.1%
Grand mean	2.002	0.146	0.021	95.0%
Missing indicator	2.002	0.146	0.021	94.9%

^1^ The unadjusted ATE is presented here for the comparison purpose; by definition, it is estimated using model$\:Y\sim T$with no covariates.

**Tabm:** Mean estimated coefficients of $\:T$, $\:{X}_{1}T$, and $\:{X}_{2}T$ and standard errors in the Secondary-Analysis model under MAR (binary covariates)

	Average Estimate	SE	MSE	95% CI coverage
$$\:T$$				
Complete case	1.649	0.245	0.184	61.3%
MI baseline-only	2.000	0.151	0.023	94.8%
MI overall	1.015	0.151	0.993	0.0%
MI by arm	1.996	0.219	0.048	91.3%
Grand mean	2.001	0.145	0.021	88.6%
Missing indicator	2.001	0.145	0.021	88.6%
$$\:{X}_{1}T$$				
Complete case	2.000	0.221	0.049	95.2%
MI baseline-only	2.176	0.205	0.073	98.2%
MI overall	1.256	0.098	0.562	0.0%
MI by arm	2.001	0.117	0.014	95.5%
Grand mean	2.355	0.125	0.141	15.9%
Missing indicator	2.199	0.293	0.125	88.2%
$$\:{X}_{2}T$$				
Complete case	1.999	0.090	0.008	95.6%
MI baseline-only	1.142	0.120	0.751	15.5%
MI overall	1.906	0.078	0.015	94.2%
MI by arm	1.994	0.071	0.005	96.2%
Grand mean	1.986	0.092	0.009	100.0%
Missing indicator	1.992	0.091	0.008	100.0%

**Tabn:** Mean estimated coefficients of $\:T$ and standard errors in the Primary-Analysis model under MNAR (binary covariates)

	Average Estimate	SE	MSE	95% CI coverage
Unadjusted^1^	1.998	0.227	0.051	95.0%
Complete case	-0.382	0.126	5.690	0.0%
MI baseline-only	2.001	0.157	0.025	96.9%
MI overall	0.332	0.125	2.796	0.0%
MI by arm	1.045	0.163	0.939	0.0%
Grand mean	2.001	0.153	0.024	94.8%
Missing indicator	2.002	0.137	0.019	94.9%

^1^ The unadjusted ATE is presented here for the comparison purpose; by definition, it is estimated using model$\:Y\sim T$with no covariates.

**Tabo:** Mean estimated coefficients of $\:T$, $\:{X}_{1}T$, and $\:{X}_{2}T$ and standard errors in the Secondary-Analysis model under MNAR (binary covariates)

	Average Estimate	SE	MSE	95% CI coverage
$$\:T$$				
Complete case	0.813	0.143	1.431	0.0%
MI baseline-only	2.000	0.156	0.024	93.7%
MI overall	0.673	0.114	1.774	0.0%
MI by arm	1.150	0.141	0.743	0.0%
Grand mean	2.001	0.153	0.023	89.4%
Missing indicator	2.001	0.137	0.019	85.1%
$$\:{X}_{1}T$$				
Complete case	2.003	0.113	0.013	95.4%
MI baseline-only	2.785	0.144	0.636	0.6%
MI overall	1.450	0.073	0.308	0.0%
MI by arm	1.739	0.080	0.075	17.6%
Grand mean	2.561	0.127	0.331	0.9%
Missing indicator	1.749	0.101	0.073	50.4%
$$\:{X}_{2}T$$				
Complete case	2.003	0.116	0.013	95.0%
MI baseline-only	1.577	0.130	0.196	85.5%
MI overall	1.950	0.081	0.009	95.9%
MI by arm	1.981	0.075	0.006	95.6%
Grand mean	2.141	0.152	0.043	97.5%
Missing indicator	1.941	0.115	0.017	99.6%

## Abbreviations

ATE: Average treatment effect
GMI: Grand mean imputation
MAR: Missing at random
MCAR: Missing completely at random
MNAR: Missing not at random
MI: Multiple imputation
MIM: Missing indicator method
RCTs: Randomized controlled trials
